# Persistent molecular remission of refractory acute myeloid leukemia with inv(16)(p13.1q22) in an elderly patient induced by cytarabine ocfosfate hydrate

**DOI:** 10.1186/s13045-014-0100-6

**Published:** 2015-02-06

**Authors:** Masahisa Arahata, Yukihiro Shimizu, Hidesaku Asakura, Shinji Nakao

**Affiliations:** Department of Internal Medicine, Nanto Municipal Hospital, 938 Inami, Nanto, Toyama 932-0211 Japan; Department of Internal Medicine (III), Kanazawa University Graduate School of Medical Science, Kanazawa, Japan; Department of Cellular Transplantation Biology, Division of Cancer Medicine, Kanazawa University Graduate School of Medical Science, Kanazawa, Japan

**Keywords:** Cytarabine ocfosfate hydrate, Acute myeloid leukemia, Inv(16)(p13.1q22), Refractory, Elderly

## Abstract

The prognosis of relapsed acute myeloid leukemia (AML) in elderly patients is dismal, even if the AML exhibits a good prognostic karyotype, such as inv(16)(p13.1q22). We present a 72-year-old female with AML with inv(16)(p13.1q22) who suffered five episodes of relapse with temporary complete remission. Maintenance chemotherapy with oral cytarabine ocfosfate hydrate eventually produced persistent molecular complete remission of her AML that had not been induced by conventional regimens including intensive chemotherapy and low dose cytarabine therapy. The high level of tolerability to oral cytarabine ocfosfate hydrate may offer elderly patients with this type of AML a good chance for a cure.

**Electronic supplementary material:**

The online version of this article (doi:10.1186/s13045-014-0100-6) contains supplementary material, which is available to authorized users.

## Introduction

Acute myeloid leukemia (AML) with inv(16)(p13.1q22) is characterized by a favorable prognosis and good response to treatment with cytarabine [1]. The strategy of treatment for AML with inv(16)(p13.1q22) is based on a series of intensive chemotherapy, which is considered more curable than prolonged maintenance chemotherapy with low-dose anti-leukemic agents even in the elderly [2-4]. On the other hand, low-dose cytarabine therapy (LDAC) is recommended for elderly patients with AML who are not considered suitable for intensive chemotherapy [5-8], but LDAC can rarely induce persistent remission [9]. Once they relapse, their prognosis is usually dismal, even if the AML is associated with inv(16)(p13.1q22) [10,11].

## Case presentation

In December 2006, a 72-year-old female was admitted to our hospital presenting with general malaise and dyspnea. A physical examination revealed anemic palpebral conjunctiva and purpura of the extremities. The complete blood count indicated severe anemia and thrombocytopenia as well as mild leukocytosis with 22.5% blast cells (Additional file 1). A bone marrow examination showed a total nucleated cell count of 41,000/μL with 67.0% blasts (Additional file 2: Figure S1A). A cytogenetic analysis of the bone marrow cells with G-banding showed 46,XX,inv(16)(p13.1q22) in all 20 dividing cells (Additional file 2: Figure S1B). Based on these findings, the patient was diagnosed as having AML with inv(16)(p13.1q22).

**Figure 1:**
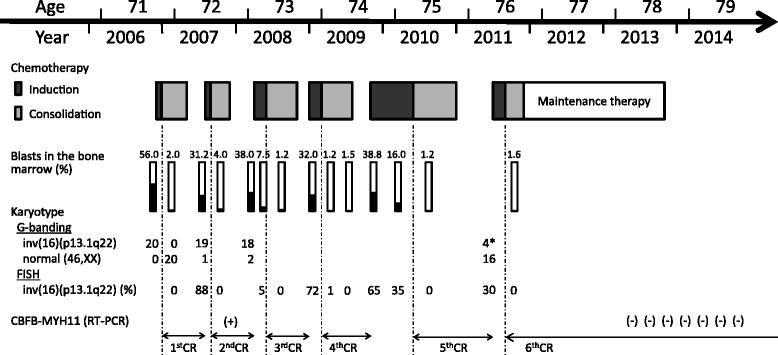
**Clinical course of the patient.** FISH: fluorescence in situ hybridization, (+): positive, (−): negative. *The karyotype was obtained from peripheral blood cells at that time because the patient rejected a bone marrow aspiration procedure.

The patient achieved complete remission (CR) after one course of induction chemotherapy comprising behenoyl cytarabine and daunorubicin according to a study protocol [12] (Table 1). However, the AML relapsed four months after the completion of the last cycle of consolidation therapy. Re-induction chemotherapy using the same regimen as the first induction induced a second CR. Thereafter, the patient suffered four further episodes of relapse with temporary remission (Figure 1). The failure to achieve durable remission even with high-dose consolidation therapy and its toxicities prompted us to select palliative care with LDAC at the third relapse. After achieving the sixth CR, the patient declined further treatment with LDAC due to toxicity. Therefore, oral cytarabine ocfosfate hydrate (SPAC) was started in order to maintain remission in November 2011. The SPAC therapy was not associated with any significant toxicity. The *CBFB-MYH11* fusion mRNA in the peripheral blood became negative after twelve courses of SPAC therapy, which was terminated in October 2013. The patient has since remained in molecular remission without chemotherapy (Figure 1).

**Table 1:** **Chemotherapy regimens and adverse events in the present case**

	**Chemotherapy**	**No.**	**Regimen**	**PS**	**BI**	Grade of adverse event*						**BW** **(kg)**	**Complications**
						**Neutropenia**	**FN or infecton**	**Anemia**	**Thrombocytopenia**	**Anorexia**	**Weight loss**		
Onset to 1st CR	Induction	1	BHAC 200 mg/m^2^ IV day 1-8	3	100	4	3	4	4	3	1	44.4	
			DNR 40 mg/m^2^ IV day 1-3										
	Consolidation	2	BHAC 200 mg/m^2^ IV day 1-5	2	100	4	3	4	4	3	1	41.6	Sepsis
			MIT 7 mg/m^2^ IV day 1-3										
		3	BHAC 200 mg/m^2^ IV day 1-5	1	100	4	3	3	3	3	1	40.8	
			DNR 25 mg/m^2^ IV day 1-2										
			ETP 100 mg/m^2^ IV day 1-3										
		4	BHAC 200 mg/m^2^ IV day 1-5	1	100	4	3	2	2	2	1	40.5	
			ACR 10 mg/m^2^ IV day 1-5										
1st relapse to 2nd CR	Induction	5	BHAC 200 mg/m^2^ IV day 1-8	4	55	4	3	4	3	3	2	39.0	Osteoporotic lumbar compression fracture Pulmonary Aspergillosis
			DNR 40mg/m^2^ IV day 1-3										
	Consolidation	6	BHAC 200 mg/m^2^ IV day 1-6	2	100	4	3	3	2	2	2	38.5	
			DNR 40 mg/m^2^ IV day 1-3										
		7	Ara-C 1 g/m^2^ IV x2 day 1-5	1	100	4	3	4	3	3	3	36.9	
2nd relapse to 3rd CR	Induction	8	LDAC day 1-14 with M-CSF day 15-28	3	100	4	3	3	4	3	3	36.5	
		9	LDAC day 1-14 with M-CSF day 1-14	2	100	4	None	3	4	3	3	34.9	
	Consolidation	10	Same as # 9	1	100	3	None	3	3	3	3	35.2	
		11	Same as # 9	1	100	3	None	3	3	3	3	36.4	
		12	Same as # 9	1	100	3	None	4	3	3	3	36.4	
		13	Same as # 9	1	100	2	None	3	3	3	2	37.5	
3rd relapse to 4th CR	Induction	14	LDAC day 1-14 with M-CSF day 1-14	1	100	4	3	3	4	3	2	37.5	
			VPA 600 mg/day PO										
		15	LDAC day 1-12 with M-CSF day 1-14	1	100	3	None	3	3	3			
			VPA 600 mg/day PO										
	Consolidation	16	Same as # 15	1	100	3	None	3	3	3	1	41.3	
		17	LDAC day 1-10 with M-CSF day 1-14	1	100	3	3	2	3	3	2	39.1	
			VPA 600 mg/day PO										
		18	Same as # 17	1	100	3	3	2	3	3	2	39.4	
		19	Same as # 17	1	100	3	None	3	3	3	1	40.6	
4th relapse to 5th CR	Induction	20	LDAC day 1-10 with M-CSF day 1-14	3	95	4	3	4	4	3	1	42.4	
		21	SPAC 200 mg/day PO day 1-14	1	95	None	None	3	None	2			
			G-CSF 100 μg SC day 1-14										
		22	Same as # 21	1	95	3	None	2	2	1	1	41.2	
		23	LDAC day 1-10 with M-CSF day 1-14	2	100	4	3	4	4	3	1	42.0	
		24	LDAC day 1-12 with M-CSF day 1-14	3	100	4	3	4	4	3	1	41.6	
		25	LDAC day 1-12 with G-CSF day 1-12	3	100	4	3	4	4	3	1	42.2	
			ACR 14 mg/m^2^ IV day 1-4										
		26	Same as # 25	2	100	3	3	3	3	3	1	41.7	
	Consolidation	27	Same as # 25	1	100	3	None	3	3	2			
		28	Same as # 25	1	100	4	None	3	3	2	2	39.6	
		29	Same as # 25	1	100	4	None	3	4	2	2	39.5	
		30	Same as # 25	1	100	4	None	3	4	2			
5th relapse to 6th CR	Induction	31	LDAC day 1-12 with G-CSF day 1-12	3	5	4	3	4	4	3	1	40.8	Depression
			ACR 14 mg/m^2^ IV day 1-4										
		32	MTX 15 mg + Ara-C 40mg + PSL 10mg IT day -1	4	5	4	3	4	4	4			Traumatic lumbar compression fracture
			LDAC day 1-10 with G-CSF day 1-12										
			ACR 14 mg/m^2^ IV day 1-4										
	Consolidation	33	LDAC day 1-10 with G-CSF day 1-12	3	5	4	3	3	3	3			
			ACR 14 mg/m^2^ IV day 1-4										
		34	Same as # 33	1	75	4	None	3	3	3	None	44.0	
		35	Same as # 33	1	90	4	None	3	4	3	None	44.6	
	Maintenance	36	SPAC 300 mg/day PO day 1-7 every 4-6 weeks	1	100	None	None	None	None	2	2	36.0	Sarcopenia

ACR: aclarubicin hydrochloride, Ara-C: cytarabine, BHAC; behenoyl cytarabine, BI: Barthel index, BW: body weight, DNR: daunorubicin hydrochloride, ETP: etoposide, FN: febrile neutropenia, G-CSF: lenograstim 100 μg subcutaneously injected or lenograstim 250 μg intravenously injected, IT: intrathecal injection, IV: intravenous injection, LDAC: cytarabine 10 mg/m^2^ subcutaneously injected twice a day, M-CSF: mirimostim 8 million units intravenously injected, MIT: mitoxantrone hydrochloride, PO: per oral, PS: performance status, SC: subctaneous injection.

*Adverse events were graded according to the Common Terminology Criteria for Adverse Events (CTCAE) version 4.03 produced by the National Cancer Institute (http://evs.nci.nih.gov/ftp1/CTCAE/About.html).

## Discussion

Our patient received lower doses of cytarabine and daunorubicin than the doses that are considered as standard doses for remission induction of AML with inv(16)(p13.1q22) nowadays, and the suboptimal doses of induction chemotherapy may be the cause of her early relapse. However, higher doses of cytarabine and daunorubicin may have put the 72-year-old woman’s life in danger due to associated toxicities. The frail woman eventually went into deep remission after maintenance therapy with a cytarabine prodrug SPAC.

SPAC has been shown to be as effective and tolerable as LDAC in treatment of AML [13-15], though its usefulness of SPAC is not well recognized because it is not available outside Japan. In this case, the AML cells were considered as highly sensitive to cytarabine because of repetitive achievement of CR induced by LDAC. Besides, SPAC was associated with fewer toxicities than LDAC (Table 1). LDAC requires the use of subcutaneous injections twice a day, but elderly patients often have difficulties visiting the hospital frequently. On the other hand, SPAC can be orally administered at home. These advantages enabled our patient to continue the maintenance therapy for two years and contributed to her persistent molecular remission. Thus, SPAC potentially offers a chance of cure for elderly patients with inv(16)(p13.1q22) without life threatening toxicities.

## Consent

Written informed consent was obtained from the patient for publication of this case report and any accompanying images. A copy of the written consent is available for review by the Editor-in-Chief of this journal.

## Additional files

Additional file 1: Table S1. Laboratory data of the patient at diagnosis. ALT: alanine aminotransferase, ALP: alkaline phosphatase, APTT: activated partial thromboplastin time, AST: aspartate aminotransferase, AT-III: antithrombin-III, CRP: C-reactive protein, γ-GTP: γ-glutamyltranspeptidase, LDH: lactate dehydrogenase, PT-INR: international normalized ratio of the prothrombin time, T-Bil: total bilirubin, T-Cho: total cholesterol. Additional file 2: Figure S1. Smear and karyogram of bone marrow aspirates. A: May-Giemsa-stained smear (x1,000). The blue arrows indicate myeloblasts and monoblasts. The percentage of eosinophils was elevated up to 16.0% of all nucleated cells. The immunophenotype of the blasts was CD2+, CD13+, CD33+, CD34+ and HLA-DR+ (data is not shown). B: Karyogram determined by G-banding. The red arrow indicates inv(16)(p13.1;q22).

## Abbreviations

AML: Acute myeloid leukemia
CR: Complete remission
LDAC: Low-dose cytarabine therapy
SPAC: Cytarabine ocfosfate hydrate
