# Supplementary material for: Persistent molecular remission of refractory acute myeloid leukemia with inv(16)(p13.1q22) in an elderly patient induced by cytarabine ocfosfate hydrate
Source: J Hematol Oncol. 2015 Feb 6;8:5. doi: 10.1186/s13045-014-0100-6 (PMC4332927; doi:10.1186/s13045-014-0100-6)
Supplement: Additional file 1: Table S1. — Laboratory data of the patient at diagnosis. ALT: alanine aminotransferase, ALP: alkaline phosphatase, APTT: activated partial thromboplastin time, AST: aspartate aminotransferase, AT-III: antithrombin-III, CRP: C-reactive protein, γ-GTP: γ-glutamyltranspeptidase, LDH: lactate dehydrogenase, PT-INR: international normalized ratio of the prothrombin time, T-Bil: total bilirubin, T-Cho: total cholesterol. [file 13045_2014_100_MOESM1_ESM.doc]

**Additional files**

**Additional file 1; Table S1.** Laboratory data of the patient at diagnosis

| Complete blood counts | | | Serum biochemical tests | | | Coagulation tests | | |
| --- | --- | --- | --- | --- | --- | --- | --- | --- |
| White blood cells | 12,000 | /µL | Albumin | 3.6 | g/dL | PT-INR | 1.85 |  |
| blast cells | 22.5 | % | LDH | 371 | U/L | APTT | 41.4 | Sec. |
| neutrophil | 4.0 | % | AST | 24 | U/L | D-dimer | 0.94 | µg/mL |
| eosinophil | 3.5 | % | ALT | 20 | U/L | Fibrinogen | 234 | mg/dL |
| basophil | 4.5 | % | ALP | 159 | U/L | Antithrombin | 81 | % |
| lymphocyte | 1.0 | % | γ-GTP | 27 | U/L |  |  |  |
| monocyte | 23.5 | % | T-Bil | 0.4 | mg/dL |  |  |  |
| Red blood cells | 170x10^4^ | /µL | T-Cho | 108 | mg/dL |  |  |  |
| Hemoglobin | 5.5 | g/dL | Creatinine | 0.68 | mg/dL |  |  |  |
| Platelets | 29,000 | /µL | CRP | 1.06 | mg/dL |  |  |  |
|  |  |  | Ferritin | 473 | ng/mL |  |  |  |

ALT: alanine aminotransferase, ALP: alkaline phosphatase, APTT: activated partial thromboplastin time, AST: aspartate aminotransferase, AT-III: antithrombin-III, CRP: C-reactive protein, γ-GTP: γ-glutamyltranspeptidase, LDH: lactate dehydrogenase, PT-INR: international normalized ratio of the prothrombin time, T-Bil: total bilirubin, T-Cho: total cholesterol.
