# Supplementary figures and images for: Persistent molecular remission of refractory acute myeloid leukemia with inv(16)(p13.1q22) in an elderly patient induced by cytarabine ocfosfate hydrate
Source: J Hematol Oncol. 2015 Feb 6;8:5. doi: 10.1186/s13045-014-0100-6 (PMC4332927; doi:10.1186/s13045-014-0100-6)

## Slide 1
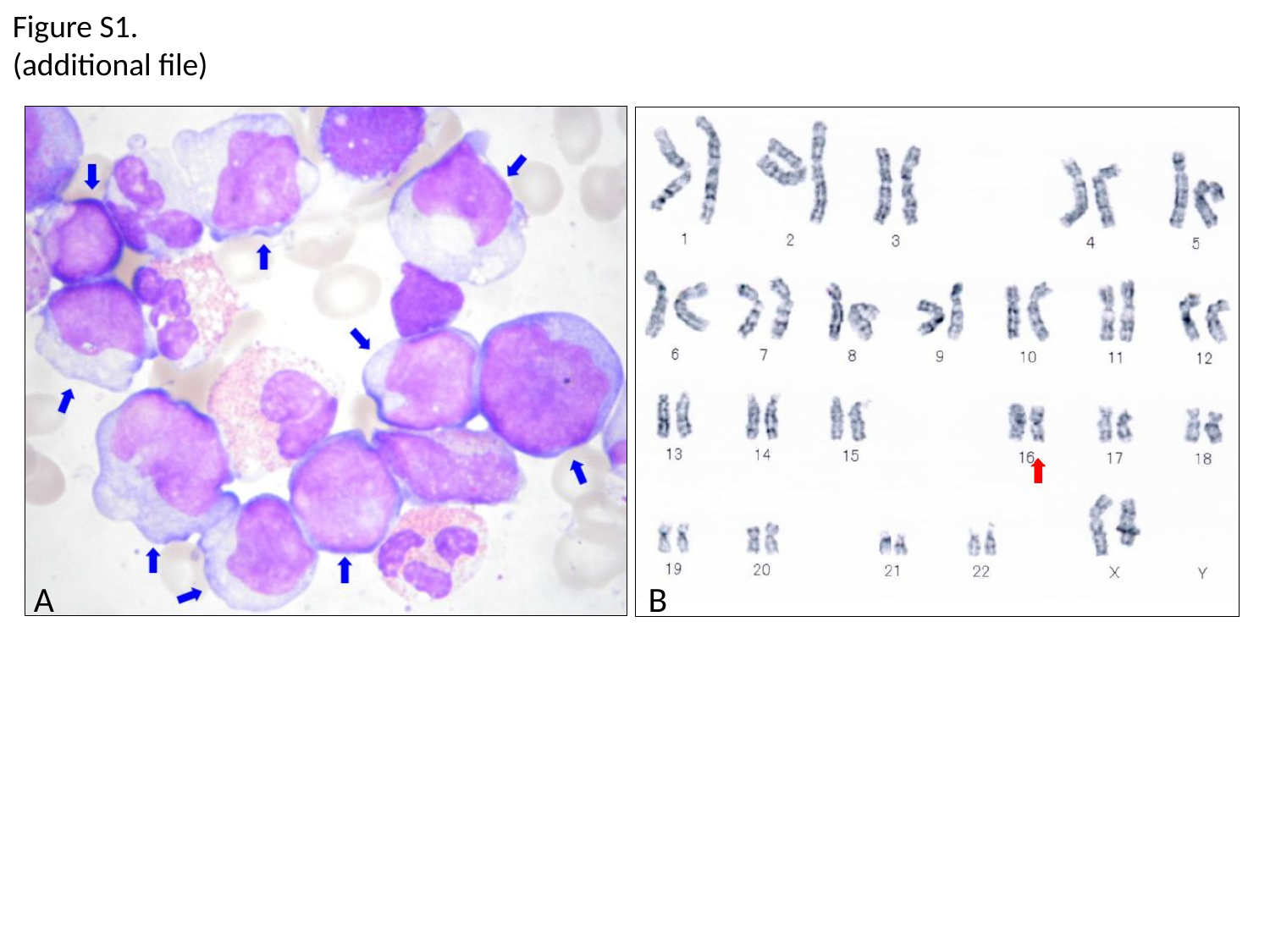

Figure S1.
(additional file)
A
B

Supplement: Additional file 2: Figure S1. — Smear and karyogram of bone marrow aspirates. A: May-Giemsa-stained smear (x1,000). The blue arrows indicate myeloblasts and monoblasts. The percentage of eosinophils was elevated up to 16.0% of all nucleated cells. The immunophenotype of the blasts was CD2+, CD13+, CD33+, CD34+ and HLA-DR+ (data is not shown). B: Karyogram determined by G-banding. The red arrow indicates inv(16)(p13.1;q22). [file 13045_2014_100_MOESM2_ESM.pptx]
